# First Confirmed Case of Canine Mortality Due to Dihydroanatoxin-a in Central Texas, USA

**DOI:** 10.3390/toxins15080485

**Published:** 2023-08-01

**Authors:** Anthea Fredrickson, Aaron Richter, Katherine A. Perri, Schonna R. Manning

**Affiliations:** 1 Lower Colorado River Authority, 3700 Lake Austin Blvd, Austin, TX 78703, USA; aaron.richter@lcra.org; 2 Department of Biological Sciences, Institute of Environment, Florida International University, 3000 NE 151st Street, North Miami, FL 33181, USAscmannin@fiu.edu (S.R.M.)

**Keywords:** benthic, cyanobacteria, dihydroanatoxin-a, anatoxin-a, *Phormidium*/*Microcoleus*, cold-water bloom, reservoir

## Abstract

The frequency of dogs becoming ill or dying from accidental exposure to cyanotoxins, produced by cyanobacteria, is increasing throughout the United States. In January and February of 2021, two dogs died and five dogs became ill after swimming in Lake Travis, central Texas, USA; one deceased dog (C_1_) was subjected to pathological testing. Algal materials, sediment samples, zebra mussel viscera, periphyton from shells, as well as fluids and tissues from the digestive tract of C_1_ were investigated for the following cyanotoxins: anatoxin-a, homoanatoxin-a, dihydroanatoxin-a (dhATX), cylindrospermopsin, saxitoxin, and microcystins. Necropsy results of C_1_ indicated neurotoxicosis with significant levels of dhATX in the duodenum tissues (10.51 ng/g dry weight (DW)), jejunum tissue (6.076 ng/g DW), and stomach contents (974.88 ng/g DW). Algae collected near the site of C_1_’s death contained levels of dhATX, ranging from 13 to 33 µg/g. By comparison, dhATX was detected at much lower concentrations in sediment samples (310.23 ng/g DW) and the periphyton on zebra mussel shells (38.45 ng/g DW). While dhATX was suspected in the deaths of canines from an event in Texas in 2019, this is the first report linking dhATX neurotoxicosis through pathological findings in Texas and potentially in the United States.

## 1. Introduction

Cyanobacteria (aka blue-green algae) are common in aquatic systems worldwide where they exist in many different forms, and some species produce harmful cyanotoxins. Cyanobacteria can be found floating in the water column (planktonic), growing on the bottom of lakes or rivers (benthic), attached to other algae (epiphytic), attached to substrates (periphyton), or in visible composites that aggregate on the water surface to form floating mats (metaphyton). These varied forms exist year-round in low numbers but can reproduce quickly in favorable conditions to form planktonic or benthic blooms [1].

During bloom events, the rapid proliferation of cyanobacteria can result in elevated levels of cyanotoxins, which can lead to animal intoxication incidents [1,2,3,4,5]. While planktonic blooms and their toxins have dominated the harmful algae literature, there is a growing need for research associated with benthic species and their toxins [6].

Abiotic factors such as climate change, invasive species colonization, and changing nutrient availability can shift energy production in a system to favor benthic growth, in a process called “benthification” [7]. Benthic cyanobacteria blooms can be cryptic, forming on the bottom of lakes and rivers attached to the rocky substrate [8]. As cyanobacterial biomass increases, oxygen bubbles produced by photosynthesis can become trapped among the cyanobacteria, causing the mats of filaments to become positively buoyant [8]. Benthic mats may become detached and float to the surface through actions such as oxygenation, wind, physical disturbance of the sediment, and/or hydrological movement. When they accumulate on the water’s surface, they are known as metaphyton mats or proliferations, and they may be pushed toward the shore, increasing the potential for exposure to humans, dogs, and other animals. Reports of dogs consuming metaphyton materials have increased in recent years [4,5,6,9]. However, reports of dog illness or death associated with mat consumption are not routinely followed by pathological testing on the animal. Therefore, it has been difficult to conclude if the cause of death was related to cyanotoxins, or more specifically benthic cyanotoxins.

On 20 February 2021, a dog (referred to as C_1_) swam in Lake Travis, located near Austin, Texas, and died from suspected neurotoxicosis. Symptoms were consistent with ingestion of cyanobacteria, i.e., trembling, inability to stand, panting, vomiting, tremor, and stumbling. While investigating the death of C_1_, there were five more reports of illness related to cyanotoxin exposure with one additional canine death reported. All dogs had been swimming in the same area where C_1_ died, and the first death (C_2_) occurred 20 January 2021.

Manning et al. [2] reported the first incidence of a dog fatality and suspected algae-related neurotoxicity in central Texas. However, the cause of death was never confirmed to be cyanotoxin-related. This report is the first to directly link the fatal consumption of metaphyton materials with dihydroanatoxin-a (dhATX), a cyanotoxin associated with the benthic species *Phormidium*/*Microcoleus*, with a dog death in Texas. The World Health Organization (WHO) does not currently have any reference values for dhATX, but a recent study showed that dhATX is roughly 3- to 4-times more toxic when ingested orally than anatoxin-a (ATX) [10]. The WHO provisional recreational water health-based reference value for ATX is 60 µg/L [11].

The current study presents data from the necropsy of C_1_, environmental cyanotoxin data found throughout the site of C_1_’s death, including the presence of cyanotoxins at the site over time, as well as additional cyanotoxin and cyanobacteria data from nearby lakes. Between the death of C_1_ and C_2_, this part of central Texas experienced an unprecedented winter storm. Beginning on 11 February 2021, temperatures ranged from 3 to −17 °C for eight days prior to the death of C_1_.

## 2. Results

### 2.1. Incident

**Figure 1:**
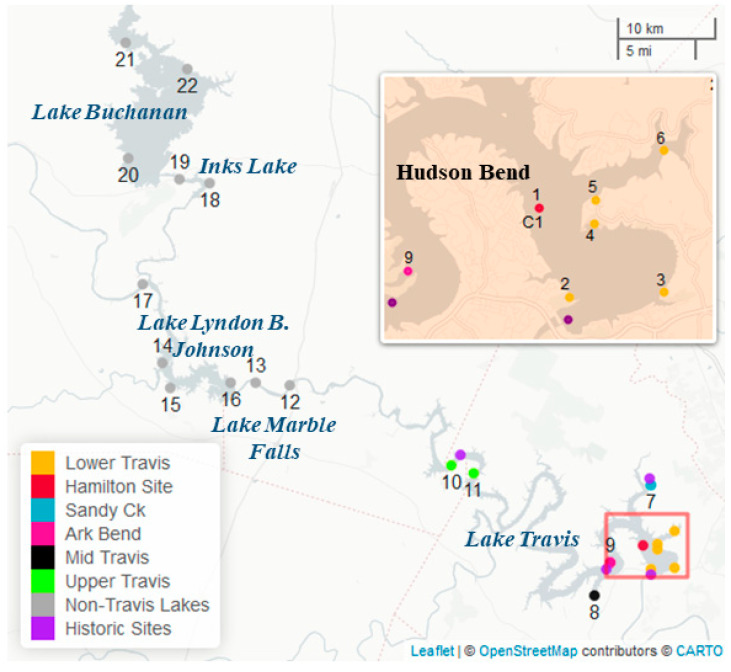
Map of the study area in central Texas, which is composed of a series of reservoirs referred to as the Highland Lakes beginning at Lake Buchanan and running south to Lake Travis, which is northwest of Austin, TX. Numbered sites correspond to locations in Table 2. Marker color within Lake Travis corresponds to the colors in the PCA plot (Figure 5) with the exception of purple markers that represent site locations where water quality data are routinely sampled.

A male golden retriever (C_1_) approximately 3 years old and weighing 51 pounds died after swimming on a freshwater reservoir (Lake Travis) located near Austin, Texas. The location, known as Hudson Bend (30.419, −97.913) (Figure 1), has water access that is restricted to private residents along the shoreline. On 20 February 2021, C_1_ swam and played along the shoreline of Hudson Bend for 3 h and 30 min. As the owner of C_1_ rinsed him off, the dog began to shake, lost the ability to walk, and began vomiting. Twenty minutes after the first signs of distress were noted, C_1_ was brought to a vet to seek medical attention. CPR was attempted, but C_1_ was ultimately euthanized. Following euthanasia, C_1_’s body was frozen and preserved until the time of necropsy on 26 February 2021.

**Figure 2:**
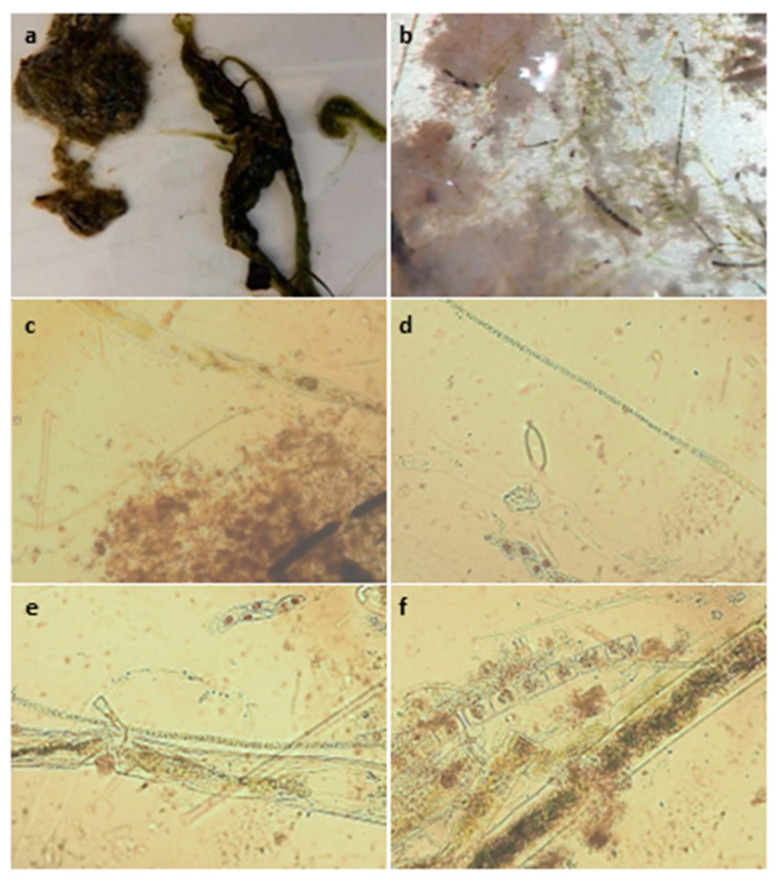
Ingesta collected from the stomach of C_1_ when examined at 1× (**a**) 50× (**b**), and 400× (**c**–**f**). Stomach contents contained several strains of microalgae, including filamentous green algae, diatoms, and cyanobacteria (not identified to species).

Once news of C_1_’s death became public, several other residents came forward to report that their dogs also experienced similar symptoms following exposure to the water and shoreline of Hudson Bend. A survey was sent to the owners who reported these symptoms, which included a report of an unconfirmed canine death that occurred on 30 January 2021. Results from this survey can be found in the corresponding data set [12]. Stomach content analysis of C_1_ showed filamentous green algae and an unidentified cyanobacteria present in the gut at the time of death (Figure 2).

**Figure 3:**
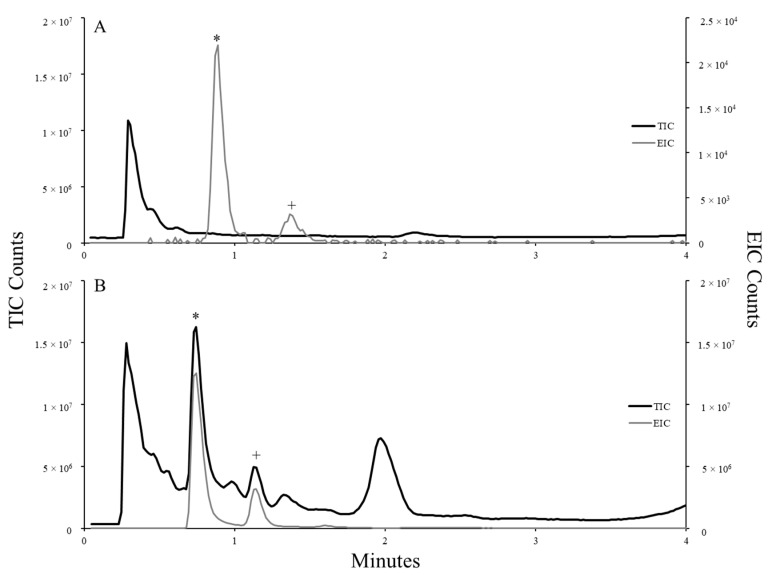
dhATX trans (*) and cis (+) isomers [M + H; mass 168.1383] as detected by HPLC-HRMS in water (**A**) and metaphyton materials (**B**). The total ion chromatogram (TIC) shows all of the analytes detected by the mass spectrometer, and peaks associated with dhATX are shown in the extracted ion chromatogram (EIC). Note the difference in scale of the second y-axis and retention time (RT, in minutes) when comparing the relative abundance of dhATX in water vs. metaphyton materials.

During the necropsy, additional samples were collected from the digestive tract and tested for cyanotoxins; dhATX concentration is reported on a dry weight (DW) basis (Table 1). A trace amount of ATX was found in C_1_ ‘s stomach content. Saxitoxin, microcystins (LA, LR, RR, YR), homoanatoxin-a (HTX) and cylindrospermopsin were all non-detect in the digestive tissues of C_1_. High levels of dhATX were found in multiple digestive tissues, including duodenum tissues (average of 10.51 ng/g DW, *n* = 2), jejunum tissue (average of 6.076 ng/g DW, *n* = 2), and from mixed stomach content (average of 974.88 ng/g DW, *n* = 2). Toxins in samples were verified by high-performance liquid-chromatography–high-resolution mass spectrometry (HPLC-HRMS) using methods described in Manning et al. 2020 [2]. Figure 3 illustrates the detection of cis- and trans-stereoisomers of dhATX in water and metaphyton samples from this study. Examples of mass spectra for dhATX are shown in Manning et al. [2] and were replicated on the same instrument in this study. The observed mass of dhATX was within 0.5 ppm of the calculated mass (168.1383 u) for [dhATX + H] ^+^, which agrees with previous results [2].

Overall, water samples had significantly lower quantities of dhATX when compared to complex extracts from metaphyton materials. Further, the retention time (RT) of dhATX in water samples was consistently observed slightly earlier in HPLC-HRMS runs when compared to extractions from more complex materials, e.g., biomass. As such, slight lags were observed for the RT of dhATX detected in extracts of metaphyton and necropsy materials. Raw and processed data from all analyses are reported in replicate and can be found in the corresponding data set [12]. The presence of cyanobacteria colonies found within the stomach content and the high levels of dhATX found in the digestive tissues strongly suggest that the cause of death for C_1_ was neurotoxicosis caused by dhATX.

### 2.2. Site Results

Composite samples containing both metaphyton and periphyton material collected from Hudson Bend on 22 February 2021, contained relatively high levels of dhATX (630 ng/mL), Composite samples containing both metaphyton and periphyton material collected from Hudson Bend on 22 February 2021, contained relatively high levels of dhATX (630 ng/mL), with low levels of microcystins/nodularins (1.13 ng/mL), saxitoxin (0.08 ng/mL), ATX (1.0 ng/mL), and non-detects for cylindrospermopsin and HTX. Results from an expanded survey around Lake Travis (analyzed only for ATX, dhATX and HTX) conducted on 3 March 2012 (*n* = 10) found dhATX present in all composite samples collected, ranging from 7.80 to 8270.00 ng/mL (Table 2 and Figure 1: gold, light grey, pink, black, and bright green locations). dhATX was detected in some water samples, whereas ATX was not detected in water samples but was present in composite samples in levels ranging from 0.80 to 13.00 ng/mL (Table 2). HTX was not detected in any of the samples collected, and therefore was omitted from Table 2. Due to the widespread occurrence of dhATX in Lake Travis, additional samples were collected during March 2021 in the upstream reservoirs (Figure 1: dark grey locations). This series of reservoirs in the study area is referred to as the Highland Lakes and includes lakes Buchanan, Inks, Lyndon B. Johnson (LBJ), Marble Falls, and Travis (two additional downstream reservoirs are sampled for cyanotoxins by the City of Austin and are not included in this study). Results showed that potentially toxigenic cyanobacteria species (PTOX) were present in all five reservoirs (Table 2) with low levels of dhATX present in Marble Falls (2.60 ng/mL) and Inks Lake (1.60 ng/mL).

**Figure 4:**
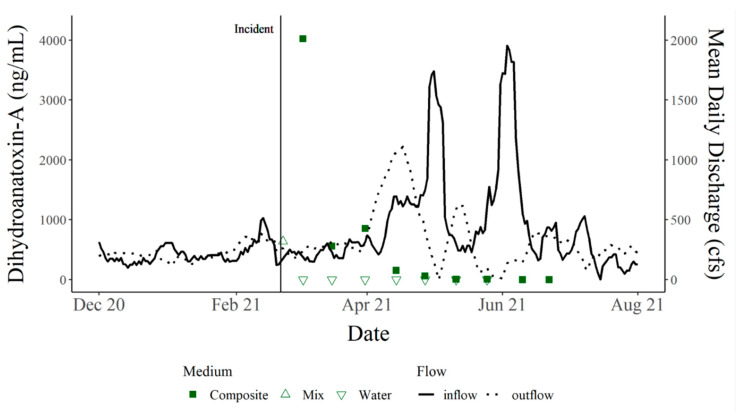
dhATX (ng/mL) and 7-day rolling average daily discharge (cfs) from the upper and lower dams on Lake Travis from December 2020 through August 2021 with the dog death occurring on 21 February 2021, represented by a vertical line. Squares represent toxins in periphyton/metaphyton composite sample while triangles represent either a water sample or a mix of metaphyton and water. dhATX in composite material was high following the incident and decreased over time with.

**Figure 5:**
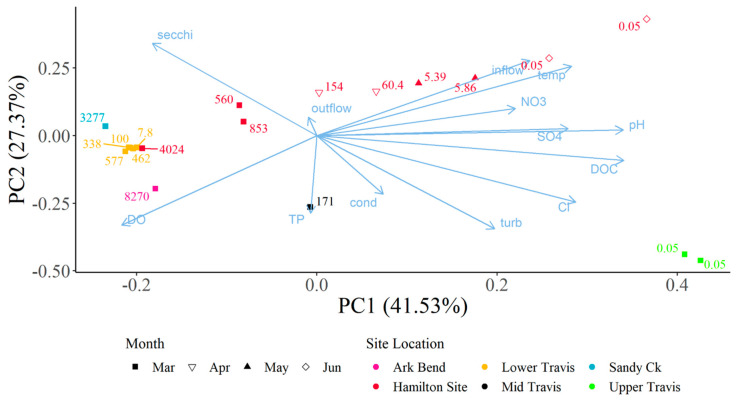
Principal component analysis ordination of water quality parameters collected from open water samples and dhATX collected from periphyton/metaphyton composite sample in Lake Travis. Marker colors correspond to site location marker colors in the site map (Figure 1) with marker shape representing the month of the sample. Marker labels represent the dhATX concentration while arrow labels represent the water quality parameter influencing the ordination. dhATX concentrations were highest with higher dissolved oxygen and secchi disc depth (higher clarity) but dh ATX concentrations were lower with reservoir inflows, nutrients, temperatures, and salts.

Continued monitoring of Hudson Bend (the site of C_1_’s death) showed that toxins were still present in metaphyton materials from 21 February 2021 through 23 June 2021, when monitoring was concluded after two measurements were below the limits of detection, i.e., non-detects (Figure 4). The levels of dhATX did not display a steady decline and instead experienced a pulse of higher toxins at the end of March before slowly declining and becoming non-detects at the end of May 2021. Principal component analysis (PCA) indicated that toxin levels around Lake Travis were highest when conditions included a higher dissolved oxygen and Secchi disc depth (higher water clarity); lower nitrate, carbon, and salt concentrations; and little inflow into Lake Travis from the upstream dam (Figure 5). At the Hudson Bend location, the PCA showed that lower toxin levels were observed with increased inflows from the upstream dam moving into the late spring and early summer. The first principal component represented 41.53% of the variation and was more heavily weighted by the inflow from the upstream dam, salts such as sulfate and chloride (surrogates for water levels in the Highland Lakes with higher salt concentrations correlating to lower lake levels), water temperature, dissolved oxygen, clarity (Secchi disc and turbidity), nitrates, and carbon. The second principal component represented 27.37% of the variation and was more heavily weighted by inflow from the upstream dam, water temperature, clarity (Secchi disc and turbidity), dissolved oxygen, and total phosphorus.

### 2.3. Water Quality Parameters

Data points collected from Hudson Bend showed several deviations from averages calculated using five years of historical water quality data collected from a downstream site (30° 23′ 31.1994″, −97° 54′ 18″) (Figure 1). Secchi disc measurements collected during the month of C_1_’s death showed levels outside the observed average over the past five years, with a return to average in April 2021.

**Figure 6:**
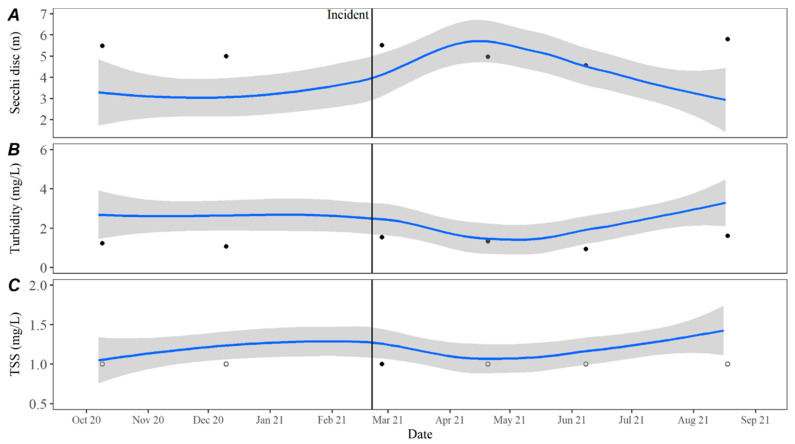
(**A**) Secchi disc depth, (**B**) turbidity, and (**C**) total suspended solids in open water samples collected at the downstream dam on Lake Travis from October 2020 through August 2021. The date of C_1_’s death is represented by the black incident line, and the blue line represents the LOESS estimate with 95% confidence intervals shown by the grey bands of historical data collected from October 2015 to October 2020. Black circles represent samples collected at the dam from October 2020 through August 2021 with open circles indicating less than the method detection limit.

**Figure 7:**
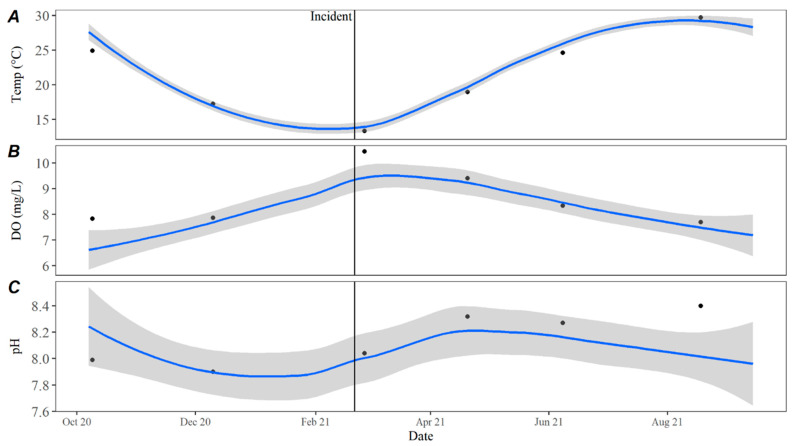
(**A**) Water temperature, (**B**) dissolved oxygen, and (**C**) pH in open water samples collected at the downstream dam on Lake Travis from October 2020 through August 2021. The date of C_1_’s death is represented by the black incident line, and the blue line represents the LOESS estimate with 95% confidence intervals shown by the grey bands of historical data collected from October 2015 to October 2020. Black circles represent samples collected at the dam from October 2020 through August 2021.

**Figure 8:**
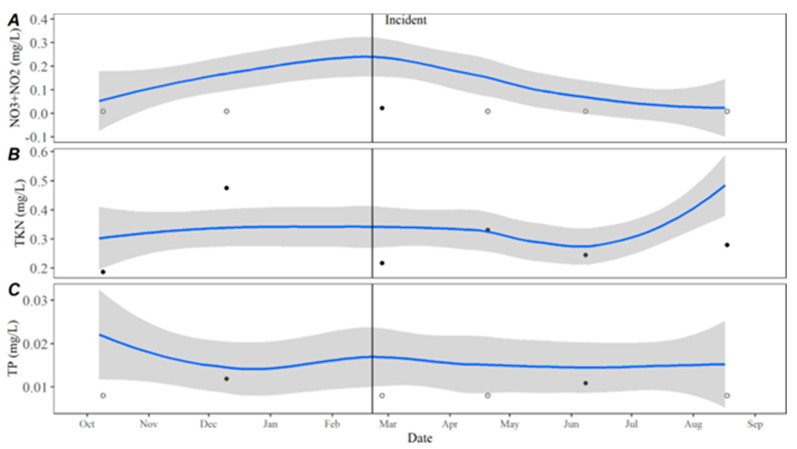
(**A**) Nitrate plus nitrite, (**B**) total Kjeldahl nitrogen, and (**C**) total phosphorus in open water samples collected at the downstream dam on Lake Travis from October 2020 through August 2021. The date of C_1_’s death is represented by the black incident line, and the blue line represents the LOESS estimate with 95% confidence intervals shown by the grey bands of historical data collected from October 2015 to October 2020. Black circles represent samples collected at the dam from October 2020 through August 2021 with open circles indicating less than the method detection limit.

**Figure 9:**
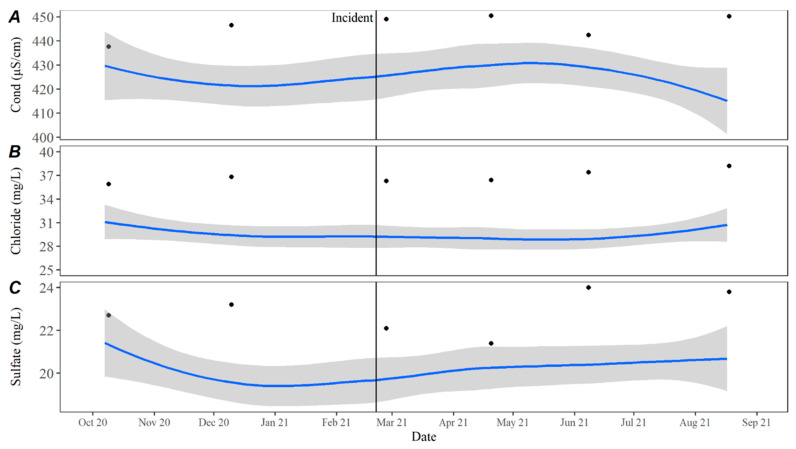
(**A**) Conductivity, (**B**) chloride, and (**C**) sulfate in open water samples collected at the downstream dam on Lake Travis from October 2020 through August 2021. The date of C_1_’s death is represented by the black incident line, and the blue line represents the LOESS estimate with 95% confidence intervals shown by the grey bands of historical data collected from October 2015 to October 2020. Black circles represent samples collected at the dam from October 2020 through August 2021.

Turbidity measurements remained consistent with the observed historical average with only a slight deviation in October 2021. Total suspended solids (TSSs) followed historical trends, Secchi disc depth was slightly higher than average, while turbidity was slightly lower than average leading up to and immediately following the incident (Figure 6). Temperature values in 2021 remained consistent with historical averages as did pH during and after the incident at Hudson Bend. Dissolved oxygen (DO) was higher immediately following the event but returned to the average after (Figure 7). Nutrient levels were lower than average immediately following the event, with nitrate and total phosphorous remaining low until June 2021 (Figure 8). Nitrates were also lower than average in December 2020, which is the closest winter sample prior to the incident. Conductivity, chloride, and sulfate levels were higher than the average for all of 2021 (Figure 9).

## 3. Discussion

Incidents of dogs becoming ill or dying after exposure to cyanobacteria have increased in recent years; however, very few of them are directly linked to cyanotoxins due to lack of supporting necropsy evidence, or incidents are reported too late to collect relevant samples [2,3]. The presence of dhATX in the stomach content and stomach tissues of C_1_ as well as the high levels found in both metaphyton/periphyton material and water samples at the site of exposure provide strong evidence that C_1_ died from dhATX neurotoxicosis. While ATX was also found at the site, recent studies have demonstrated that dhATX is more toxic than ATX when ingested orally with a lethal dose of 8 mg/kg for dhATX and 25 mg/kg for ATX [10].

By nature, benthic cyanobacteria can be cryptic, making bloom detection difficult and expensive to monitor. Often, cyanobacteria and cyanotoxin monitoring efforts occur during the high-risk seasons for planktonic blooms of summer and fall to curb costs. Survey results collected from residents of Hudson Bend indicate that dogs were becoming ill prior to the death of C_1_ and before the onset of the winter storm that impacted Texas (corresponding data [12]). This information suggests that cyanotoxins and the cyanobacteria producing them were present before the cold temperatures and persisted afterward. Our results have exposed a need to monitor cyanotoxins year-round to accurately categorize the presence/absence of cyanotoxins each season. The persistence of dhATX is supported by the continued monitoring of Hudson Bend that took place following the incident, ending only when two non-detects of dhATX occurred in a row. Additionally, this study indicates that the site of canine intoxication or death may remain toxic for months after the initial event, which could be a result of low flows in the system. Previous work has demonstrated a correlation of low flow and the colonization of benthic mats [13]. While our study did not quantify mat presence, our PCA shows a correlation between increasing flows and a subsequent decrease in toxin levels. The literature focuses on the presence and abundance of cyanotoxins at the time of the dog’s death, but it remains unclear whether follow-up monitoring was conducted following the incidents [2,6,9,14].

The survey of Lake Travis showed that high levels of dhATX can be present in mat material, while whole water samples collected directly next to mats can be non-detects for dhATX. This observation supports previous work suggesting that consumption of lake water alone is often not enough to cause intoxication events, as cyanotoxins can have low stability in the water matrix, and cyanotoxin-related deaths are most likely caused by consumption of mat material [5,14]. Additionally, the presence of dhATX while ATX is non-detect demonstrates the importance of identifying the genus or species of cyanobacteria present in a sample to identify which cyanotoxin analytical tests should target. Some may consider dhATX a degradation product of ATX, but recent work has suggested that it can be present intracellularly within certain cyanobacteria species [10]. This suggests that dhATX may occur independently of ATX; therefore, screening methods for cyanotoxins should not rule out the presence of dhATX based on the absence of ATX.

Water quality parameters collected from Lake Travis before, during, and after the dhATX exposure event can shed light on how these events may be impacted by water quality. While water and ambient air temperatures were unusually cold in the week leading up to C_1_’s death, historical routine water quality data in Lake Travis showed that water temperatures in the months before, during, and after the death of C_1_ were consistent with observed trends from the previous five years. Reinl et al. 2023 defined a cold water cyanobacterial bloom as a cyanobacterial bloom that occurs in freshwater lakes when water temperatures are below 15 °C [15]. In the week leading up to the death of C_1_, ambient air and water temperatures in Texas were historically low (<15 °C), with an extreme temperature difference potentially leading to turnover in Lake Travis. Physical disturbances or extreme storms can disturb the benthos and cause benthic proliferations to float to the surface [8,15], which could explain the sudden onset of benthic material along the shorelines of Lake Travis.

The decrease in turbidity and increase in secchi depth values in Lake Travis over the past five years may be a result of the continuing invasion and colonization by zebra mussels, *Dreissena polymorpha*. Zebra mussels were first detected in Lake Travis in 2017 [16] and have since colonized and maintained a breeding population (Lower Colorado River Authority, unpublished data). Established zebra mussel colonies can lead to benthification within a system through enhanced water clarity that allows greater benthic photosynthesis opportunities and increased deposition of nutrients to the benthos to support more biomass [7]. A review by Vadeboncoeur et al. [17] demonstrated that benthic algal proliferations can specifically benefit from an increase in zebra mussel presence due to their ability to capture nutrients from the water column and deposit them into the benthos. Another study found that inland lakes in Michigan with established zebra mussel populations had around 3.3 times higher microcystin levels when compared to uninvaded lakes [18]. The established presence of a reproducing zebra mussel population in Lake Travis could explain why benthic proliferations of cyanobacteria are becoming more common.

## 4. Conclusions

Necropsy evidence coupled with environmental samples containing dhATX provide strong evidence that C_1_ ultimately died from dhATX neurotoxicosis. In addition to confirming the first canine death related to dhATX in Texas, this study supports the growing body of literature concerning not only the rise in benthic cyanobacterial blooms but provides evidence that benthic blooms can occur in winter following extreme temperature shifts. Since the event of C_1_’s death in Lake Travis, the managing authority over the waterbody has implemented a year-round monitoring program for common benthic and planktonic cyanotoxins. However, there is little to no guidance for monitoring programs regarding benthic proliferations, with WHO threshold values only applying to whole water samples and no such guidelines for dhATX in water or metaphyton materials. This lack of recreational threshold guidelines for benthic proliferations can make lake and river closures difficult to justify. Therefore, development of actionable guidelines for dhATX is a crucial next step to maintain public health and help inform waterbody management strategies.

## 5. Materials and Methods

### 5.1. Study Site

Lake Travis (30.42° N, 97.91° W) is an impoundment of the Texas Colorado River created after the construction of Mansfield Dam was completed in 1942. It is a water supply reservoir and is the fifth reservoir in the Highland Lakes chain. Hudson Bend is the most downstream major bend in Lake Travis. Residence time and water levels in Lake Travis fluctuate based on inflows and management of the reservoir according to a stakeholder-driven and state-approved water management plan. The Lower Colorado River Authority collects routine water quality data from Lake Travis and the Texas Colorado River basin as a partner in the Texas Clean Rivers Program (CRP) administered by the Texas Commission on Environmental Quality (TCEQ) according to Texas Water Code, Section 26.0135. Historical data used to identify five-year trends in Lake Travis were collected from a CRP site located in front of Mansfield Dam (30° 23′ 31.1994″, −97° 54′ 18″).

### 5.2. Sample Collection and Analysis

Whole water samples were collected at the surface (approximately 0.3 m depth) and contained no proliferations or algal material. Composite samples consisted of any floating mats, or epiphytic and/or periphytic algal material found at the site. Both whole water and composite samples were collected in amber bottles and frozen until processed. Historic water quality data from Lake Travis were collected following protocols outlined by the TCEQ Surface Water Quality Monitoring Procedures, Volume 1 [19]. Potentially toxigenic cyanobacteria were identified by GreenWater Laboratories using a Nikon TE200 inverted microscope equipped with phase contrast and epifluorescence (green light excitation, 510–560 nm). Samples were prepared as wet mount slides and scanned at 400× and 100× to narrow down which cyanobacteria were present and determine which cyanotoxins would be tested for. Full microscopy methods and photos of PTOX cyanobacteria from this analysis can be found in the corresponding data set [12].

### 5.3. Animal Necropsy and Histopathology

A full necropsy of C_1_ was performed by Texas A&M Veterinary Medical Diagnostic Laboratory, which included examining the musculoskeletal, respiratory, circulatory, digestive, hepatobiliary, hemolymphatic, urinary, reproductive, endocrine, and nervous systems. Necropsy included a toxicology screen for a broad range of organophosphates, some carbamates and organochlorine insecticides, as well as permethrin, petroleum products, and some drugs. Necropsy also included a screen for botulism A, B, and C. Twenty-three tissue samples were collected for histopathology. The full necropsy report can be accessed in the corresponding data set [12]. Additional digestive tissues (jejunum, duodenum, and stomach content fluid) were reserved for high-performance liquid-chromatography–high-resolution mass spectrometry (HPLC-HRMS).Stomach contents, fluids, and tissue samples were analyzed by UC Davis Veterinary Medicine using liquid chromatography (LC) coupled with mass spectrometry (MS) (see report in corresponding data [12]). Microcystins (LA, LR, RR, YR) and cylindrospermopsin were analyzed using LC-MS/MS and STX and ATX were analyzed using LC-MS/MS/MS. Additional digestive tissues (jejunum, duodenum, and stomach content fluid) were reserved for HPLC-HRMS to analyze for dhATX. The full necropsy report can be accessed in the corresponding data set [12].

### 5.4. Toxin Analysis

Water samples and composite metaphyton/periphyton material samples were analyzed by GreenWater Laboratories, sample analysis methods are described in the corresponding data set, and the full reports can be viewed [12]. Environmental and necropsy samples were extracted, enriched, and prepared for biochemical analysis. Extraction and enrichment steps were performed in dimmed lighting, and sample tubes were wrapped in foil to minimize photolysis. Prior to processing, samples were centrifuged for 5 min at 2100× *g* to sediment debris. Water and solid/biomass extracts were processed by C18 solid-phase chromatography to enrich cyanotoxins and analyzed by HPLC-HRMS, according to procedures described in Manning et al., 2020 [2]. Sediments (15 g) were extracted using double-distilled water (ddH_2_O) with sonication. Zebra mussels (six bodies) were shucked and extracted in ddH_2_O with 1% HCl (Fisher Chemical, Fair Lawn Industrial Park, Fair Lawn, NJ, USA) assisted by heating (60 °C) and sonication for 6 h; and 12 zebra mussel half shells were extracted in ddH_2_O for 2 h with sonication. Necropsy liquids and tissues were extracted in methanol and 1% HCl with sonication for 6 h. Extracts were dried and resuspended in 5% MeOH in preparation for analysis by HPLC-HRMS. All samples were performed in duplicate.

Clarified lake water (50 mL) and extracts (25 mL) were passed over Waters Sep Pak C18 solid-phase extraction (SPE) cartridges (Waters Corporation, Milford, Massachusetts, USA) to enrich targeted compounds. SPE cartridges were activated with 5 mL of 100% MeOH (Fisher Chemical, Fair Lawn Industrial Park, NJ, USA) and conditioned using 5 mL of ddH_2_O. Then, the water or algal sample supernatant was slowly pushed through the column to adsorb metabolites. The eluate was retained as the sample load, and 5 mL of 100% MeOH was pushed through the cartridge to collect metabolites adsorbed on the column. Methanolic fractions were dried under nitrogen at 40 °C in pre-weighed glass vials. Once dried, extract residues were resuspended in 1 mL of 5% MeOH (aqueous), representing a 25- to 50-times enrichment from the original samples. All samples were prepared for analysis containing 0.1 ng/µL of caffeine internal standard (Fisher Chemical, Fair Lawn Industrial Park, NJ, USA) to monitor injection consistency and detection sensitivity; 100 µL of each sample with standard was transferred to a glass insert in a 2 mL amber glass vial in preparation for HPLC-HRMS.

Samples were analyzed on an Agilent 6530 QTOF instrument (Agilent, Santa Clara, CA, USA). Samples (10 µL) were injected and separated on an Agilent Zorbax Eclipse Plus C18 column (50 mm length, 2.1 mm ID, 5 µm particle size, packed; Santa Clara, CA, USA) using a water:methanol gradient with 0.1% formic acid addition (Fisher Chemical, Fair Lawn Industrial Park, Fair Lawn, NJ, USA). The gradient was ramped from 1.0 to 95.0% MeOH over 12 min and held at 100% MeOH for 6 min. Mass spectrometry data were collected following electrospray ionization (ESI) operated in positive mode to produce the total ion chromatogram (TIC) and related extraction ion chromatograms (EIC) for target toxins.

Due to an absence of commercial reference materials, quantitative analysis of dhATX in samples was estimated using the caffeine internal standard as a proxy analyte of similar mass and polarity. The area underneath each peak was combined to represent the quantity of dhATX present in each injection and extrapolated to estimate the amount of dhATX per L or water or per g or biomass sampled (*n* = 2).

### 5.5. Statistical Analysis of Water Quality Parameters

Principal Component Analysis (PCA) was used to assess composite sample dhATX concentrations alongside multiple water quality parameters collected in open water locations within Lake Travis using the prcomp function in the R Statistical Software (v4.3.0; R Core Team) [20]. Toxins from algal samples were collected from 3 March 2021 to 22 June 2021. Water quality parameters were collected as part of the Texas Clean Rivers Program (CRP) in lower Lake Travis, the Sandy Creek arm, the middle of Lake Travis, near Arkansas Bend, and in upper Lake Travis. Parameters collected and used within the PCA include specific conductivity, dissolved oxygen, water temperature, pH, turbidity, inflow from the upstream dam, outflow from the downstream dam, secchi disc depth, nitrate, chloride, sulfate, dissolved organic carbon, and total phosphorus. Composite samples collected at sites 1, 2, 3, 4, 5, and 6 corresponded to water quality data collected in lower Lake Travis (30.3920° N, 97.9050° W; 12,302); those at site 7 corresponded to data in the Sandy Creek arm (30.4786° N, 97.9056° W; 12,307); those at site 8 corresponded to data in the middle of Lake Travis (30.3731° N, 97.9636° W; 15,428); those at site 9 corresponded to data near Arkansas Bend (30.3960° N, 97.9510° W; 12,309); and those at sites 10 and 11 corresponded to data in upper Lake Travis (30.4990° N, 98.1040° W; 12,316).

Composite samples were not collected on the same day as the water quality samples, so water quality parameters were imputed and estimated based on mathematical formulas, using equations for Julian day. The formulas were derived from water quality samples collected every other month at each CRP site from 25 February 2021 to 14 December 2021, for a total of six samples at each site. The frequency of observed values would not allow for the evaluation of acute changes to water quality, but it is appropriate to allow the authors to evaluate general trends of water quality within the site location during the event. Quadratic to quintic equations were chosen as imputation equations for each parameter and site based on the fit of the equation. The R^2^ values of the imputation equations ranged from 0.1537 to 1.0; however, 54% of the equations had an R^2^ value above 0.9 and an additional 27% of the equations had an R^2^ over 0.7. The majority of the imputation equations fit the data well with only a few instances where no equation fit the data well.

Time series averages and 95% confidence intervals for water quality parameters were constructed in lower Travis based on data collected from October 2015 to October 2020. Samples were typically collected throughout each year in February, April, May, June, July, August, September, October, and December. For all parameters except TKN, the total sample size for computing the time series averages was *n* = 46. There were quality control issues with one TKN sample and the sample size for TKN was *n* = 45. The average for each parameter by day was calculated by transforming the date to Julian day and applying locally estimated scatterplot smoothing (LOESS [21]), also known as local polynomial regression, to the collected data using the LOESS function in the R Statistical Software R Statistical Software (v4.3.0; R Core Team 2023) [20]. Using LOESS allows for flexibility in fitting the model by using localized subsets rather than a global formula, which allows the average to move throughout the year based on seasonal environmental conditions. Water quality data collected from October 2020 to September 2021 were compared to the averages to evaluate how conditions directly prior to, during, and after the incident compared to historically collected data.

## Figures and Tables

**Table 1:** Environmental samples including periphyton/metaphyton composites, sediment, zebra mussel (*Dreissena polymorpha*) viscera, and zebra mussel shell periphyton were collected from Hudson Bend two days after the death of C_1_. Results from samples of C_1_’s digestive tissues are reported below. Samples were analyzed in replicates and averages are reported (*n* = 2).

Date	Sample Source and Type	dhATX (ng/g)
	**Hudson Bend**	
22 February 2021	Water	0.29
22 February 2021	Water + Algae	2549.92
22 February 2021	Algae	12,047.15
24 February 2021	Sediments	420.16
24 February 2021	Zebra Mussel Viscera	n.d.
24 February 2021	Zebra Mussel Periphyton	49.42
	**Canine**	
24 February 2021	Duodenum Tissue	10.51
4 March 2021	Duodenum Fluid	47.88
4 March 2021	Jejunum Tissue	6.076
4 March 2021	Jejunum Fluid	18.59
4 March 2021	Stomach Content	974.88

n.d. = Non-detect = levels below 0.05 ng/g.

**Table 2:** Results from a one-time survey for cyanotoxins in the Highland lakes including Lake Travis (1–11), Lake Marble Falls (12–13), Lake LBJ (14–17), Inks Lake (18–19), and Lake Buchanan (20–22). Non-detect (n.d.) = toxin levels below 0.05 µg/L.

Site	Sample Type	Sample Date	ATX ng/mL	DhATX ng/mL	PTOX Species Identified
1	Water Composite	3 March 2021	n.d. 0.80	n.d. 100.00	*Phormidium*/*Microcoleus* spp.
2	Water Composite	3 March 2021	n.d. 0.80	n.d. 100.00	*Phormidium*/*Microcoleus* spp.
3	Water Composite	3 March 2021	n.d. 0.90	n.d. 7.80	*Phormidium*/*Microcoleus* spp.
4	Water Composite	3 March 2021	n.d. 1.90	0.15 462.00	*Phormidium*/*Microcoleus* spp., *Oscillatoria* spp., *Geitlerinema*/*Anagnostidinema* sp.
5	Water Composite	3 March 2021	n.d. 1.50	n.d. 338.00	*Phormidium*/*Microcoleus* spp., *Oscillatoria* sp.
6	Water Composite	3 March 2021	n.d. 1.80	n.d. 577.00	*Phormidium*/*Microcoleus* spp., *Oscillatoria* sp., *Geitlerinema*/*Anagnostidinema* sp.
7	Water Composite	3 March 2021	n.d. 6.80	0.06 3277.00	*Phormidium*/*Microcoleus* spp.
8	Water Composite	3 March 2021	n.d. 0.80	n.d. 171.00	*Phormidium*/*Microcoleus* spp., *Oscillatoria* sp.
9	Water Composite	3 March 2021	n.d. 13.00	0.15 8270.00	*Phormidium*/*Microcoleus* spp., cf. *Oscillatoria* sp., *Geitlerinema* sp.
10	Water Composite	17 March 2021	n.d. n.d.	n.d. n.d.	*Phormidium*/*Microcoleus* spp., *Geitlerinema* sp.
11	Water Composite	16 March 2021	n.d. n.d.	n.d. n.d.	*Oscillatoria* sp., *Geitlerinema* sp.
12	Water Composite	16 March 2021	n.d. n.d.	n.d. n.d.	*Planktothrix* sp.
13	Water Composite	16 March 2021	n.d. 1.60	n.d. 2.60	*Phormidium*/*Microcoleus* sp.
14	Water Composite	16 March 2021	n.d. n.d.	n.d. n.d.	None
15	Water Composite	16 March 2021	n.d. n.d.	n.d. n.d.	None
16	Water Composite	16 March 2021	n.d. n.d.	n.d. n.d.	*Phormidium*/*Microcoleus* sp.
17	Water Composite	16 March 2021	n.d. n.d.	n.d. n.d.	None
18	Water Composite	17 March 2021	n.d. 0.20	n.d. 1.60	*Phormidium*/*Microcoleus* spp.
19	Water Composite	17 March 2021	n.d. n.d.	n.d. n.d.	*Phormidium*/*Microcoleus* sp., *Dolichospermum* sp.
20	Water Composite	17 March 2021	n.d. n.d.	n.d. n.d.	*Microcystis* sp., *Dolichospermum* sp.
21	Water Composite	17 March 2021	n.d. n.d.	n.d. n.d.	*Phormidium*/*Microcoleus* spp., *Dolichospermum* sp.
22	Water Composite	17 March 2021	n.d. n.d.	n.d. n.d.	*Phormidium*/*Microcoleus* spp., *Dolichospermum* sp.

## Data Availability

The data that support this research are openly available on Mendeley Data at https://doi.org/10.17632/nn6vt666vx.1.
